# Zimbabwean youth with disabilities in Technical Vocational Education and Training: An institutional ethnography

**DOI:** 10.4102/ajod.v15i0.2030

**Published:** 2026-08-24

**Authors:** Mahara Goteka, Nyaradzai E. Munambah, Lana van Niekerk, Lieketseng Ned

**Affiliations:** 1 Division of Disability and Rehabilitation Studies, Department of Global Health, Faculty of Medicine and Health Sciences, Stellenbosch University, Cape Town, South Africa; 2 Department of Physiotherapy and Occupational Therapy, School of Allied Health, Faculty of Health Sciences and Veterinary Medicine, University of Namibia, Windhoek, Namibia; 3 Division of Occupational Therapy, Department of Health and Rehabilitation, Faculty of Medicine and Health Sciences, Stellenbosch University, Cape Town, South Africa

**Keywords:** disability inclusion, institutional ethnography, TVET, youth with disabilities, Zimbabwe, Listening Guide

## Abstract

**Background:**

People with disabilities remain under-represented in Technical and Vocational Education and Training (TVET), despite global and regional policies promoting their inclusion.

**Objectives:**

This study examined the barriers and facilitators to the inclusion of youth with disabilities in Zimbabwe’s TVET programmes.

**Methods:**

Situated in a critical qualitative paradigm, we utilised an institutional ethnographic (IE) design to explore how youth with disabilities experienced inclusive TVET. Data were generated through observations and semi-structured interviews with 11 youth with disabilities and two principals at inclusive centres. We used IE and the Listening Guide to map how institutional policies, practices and social norms coordinate learners’ participation and lived experiences.

**Results:**

Participation in TVET was influenced by intersecting institutional and social factors. Facilitators encompassed positive instructor conduct, inclusive pedagogy, robust family and community support, favourable societal attitudes and accessible infrastructure. Conversely, participation was hindered by limited government support, caregiver fatigue, families’ low socioeconomic status, disability type, literacy requirements, inaccessible infrastructure, long distances and conflicts between social norms and institutional rules.

**Conclusion:**

Achieving inclusive TVET in Zimbabwe requires aligning instructor capacity, accessible foundational education and sustainable government funding to mitigate families’ weak socioeconomic status.

**Contribution:**

Beyond identifying barriers and facilitators, this study offers a novel methodological contribution, unravelling the invisible, text-mediated forces that coordinate learners with disabilities’ experiences across pre-enrolment, training and post-training work transitions. It provides practitioners and policymakers in low-resource settings with a user-focused mapping of how institutional mechanisms can be reconfigured to foster authentic inclusion.

## Introduction

Despite international, regional and national legal and policy decrees that promote the inclusion of people with disabilities in Technical Vocational Education and Training (TVET) programmes, they remain significantly under-represented. In most countries, TVET attendance rates are substantially lower among people with disabilities than among those without (International Labour Organization 2025). In low-income countries, only one in 10 people with disabilities have access to TVET (International Labour Organization 2017).

Technical Vocational Education and Training imparts knowledge and skills for occupations (Moodie 2002; Power 1999), preparing human resources for the labour market. Its ability to alleviate poverty (Malle, Pirttimaa & Saloviita 2015) by preparing a person to work and earn income positions it as part of the solution to inequality, unemployment and poverty among youth with disabilities (Karim & Gide 2018; Muzite 2021). Its role in poverty alleviation is adequately reflected in the growing global calls to ensure its accessibility for persons with disabilities. Article 24.5 of the United Nations Convention on the Rights of Persons with Disabilities (UNCRPD) calls upon State Parties to ensure that people with disabilities can have equitable access to TVET (United Nations 2006). Sustainable Development Goal 4 highlights the importance of TVET and the need to ensure its accessibility to persons with disabilities. Though these instruments are crucial, without complementary national-level policy and legal frameworks, they are insufficient to enforce the rights of persons with disabilities (Lang 2009). Thus, several low- and middle-income countries (LMICs) have ratified the Convention and translated it into local laws and policies. According to The World Bank (2026), LMICs refer to low-income countries with a Gross National Income (GNI) below USD 1135 and lower-middle-income countries with a GNI per capita between USD 1136 and 4495 according to its classification of economies. Gross National Income per capita is a measure of the total income earned by people in a particular nation, including investment income and remittances from citizens working abroad, averaged per person (The World Bank 2025).

Zimbabwe has made some progress over the past two decades regarding the inclusion of persons with disabilities in mainstream development. Its government ratified the UNCRPD in 2013 and since then, has made significant legislative and policy commitments. For instance, Section 83 of Zimbabwe’s 2013 Constitution laid a strong foundation for inclusive development broadly and in particular, compels the Government to provide accessible educational facilities and fund the education and training needs of persons with disabilities (United Nations Educational, Scientific and Cultural Organization [UNESCO] 2021). The Zimbabwe National Youth Policy 2020–2025 emphasises the need for an enabling environment and accessible infrastructure for TVET institutions. The Zimbabwe National Disability Policy of 2021 outlines comprehensive measures to ensure participation of persons with disabilities in TVET, including training fee exemptions, adapting training facilities to make them accessible, curriculum reform, mechanisms to ensure availability of assistive devices, training of instructors, setting up of disability resource centres at TVET institutions and measures to establish and strengthen linkages between TVET and employers. Despite this progress, persisting systemic and operational barriers continue to hinder the participation of persons with disabilities in Zimbabwe’s TVET programmes. The Ministry of Higher and Tertiary Education, Innovation, Science and Technology Development and the Ministry of Youth Empowerment, Development that implement TVET do not have inclusive education policies (UNESCO 2021).

While facilitators and barriers to inclusive TVET have been studied extensively, evidence on practices that improve the experiences of youth with disabilities who participate in TVET programmes remains lacking (Makwembere 2023). At the regional level, the 2008–2009 UNESCO International Project on Technical and TVET conferences in Mozambique and Eswatini highlighted the lack of exemplary inclusion practices in TVET across Africa, emphasising the need to continuously identify and share best practices. Responding to this call, this study aims to gather the experiences of youth with disabilities, identify success factors for their inclusion, and pinpoint barriers to their enrolment and participation.

### Theoretical framework

This study is guided by Bronfenbrenner’s 1979 ecological framework, which explains human development through the interaction between individuals and their environments (Bronfenbrenner 1994). The analysis considers four interconnected systems: the micro-system of participants’ immediate experiences at home and school; the meso-system examining interactions between settings, such as home and community; the exo-system involving external influences, such as workplace practices; and the macro-system with broader laws, policies and cultural beliefs that shape others. Accordingly, this study analyses how policy environments interact with daily realities in schools, communities and homes, thereby affecting the TVET experiences of youth with disabilities.

## Research methods and design

### Study design

This study explored the success factors and barriers to youth with disabilities’ inclusion in TVET from the lived experiences of those who had participated in an inclusive TVET programme. Consistent with the inductive approach inherent to an ecological framework, it applied Dorothy Smith’s Institutional Ethnography (IE), a critical qualitative methodology that starts its analysis from the perspective of people’s everyday local experiences (Kearney et al. 2019), allowing researchers to focus on what people do and how this is organised by institutional texts (e.g. rules, regulations and policies) and the social organisation emanating from them (Petrovic et al. 2015). Institutional Ethnography enables analysing how institutional text-mediated arrangements, including policies, rules, guidelines, standards and laws, influence the experiences of youth with disabilities who participate in a TVET programme.

### Study setting

Technical Vocational Education and Training in Zimbabwe – regulated by the *Manpower Planning and Development Act 28:02 of 1984* (amended in 1996) – is delivered through secondary schools and public and private training centres, which are owned by the government, non-governmental organisations (NGOs), faith-based organisations, private individuals or corporations. Accordingly, this study was conducted at two inclusive TVET centres in Zimbabwe’s Mashonaland Central Province.

The two centres were part of the ‘TVET for All’ project, funded by a consortium of NGOs. One of the sampled training centres is owned and run by an NGO, and the other is a government institution under the Ministry of Youth Empowerment, Development and Vocational Training.

### Sampling and recruitment

Eleven learners with disabilities (L01–L11), who had either currently enrolled in or completed a 6-month vocational course at two sampled centres, were selected for the study using purposive sampling. The principals of the two centres (SP01–SP02) participated as key informants to triangulate the participant learners’ accounts. Participant learners were initially identified from lists obtained from both centres using snowball sampling to supplement recruitment, as some former learners were unreachable. Table 1 presents the participants’ profiles, including their demographics.

**TABLE 1:** Participant profiles disaggregated by gender, age group and distance (*N* = 11).

Variables	Category	Count
Gender	Male	5
	Female	6
	Other	0
Age group (years)	18–22	3
	23–27	7
	28–32	1
	33–35	0
Distance from home to training centre (km)	Within 5	1
	5–10	1
	11–15	0
	More than 15	9

Table 2 presents the nature of the impairments represented by the learner participants.

**TABLE 2:** Impairment types represented among learner participants (*N* = 11).

Impairment type	Count
Cerebral palsy	1
Wheelchair user	1
Dwarfism	2
Speech and hearing impairment	2
Amputee	1
Other physical disabilities	4

### Data collection

The first author collected data from December 2023 to March 2025 through observations and semi-structured interviews, each lasting 15–60 min and following interview guides built around the ecological framework. The guide for youth with disabilities focused on four main topics: participant experiences in the training programme (with probes on accessibility, transport, accommodation, support services, learning environment, instructors, social interactions and policies); perceptions of training outcomes; the influence of family, vocational school, community and policy on these outcomes; and recommendations to improve the participation of youth with disabilities. Recruitment continued until no new thematic categories emerged from the narratives. After the 11th interview, the interviewer noted repetition in the narrative patterns, so decided to halt participant recruitment, and subsequently interviewed the principals to validate some of the accounts from learner participants.

Follow-up interviews with the two centres’ principals further confirmed institutional traces and texts (policies, rules and regulations) identified through ethnographic analysis of the participants’ interviews.

All recruited participants consented to participate in the study and allowed audio recordings of their interviews. Consent forms were translated into the participants’ local languages to ensure clarity. There was a sign language interpreter for the two participants with hearing and speech impairments.

The data collection process observed key ethical considerations, such as anonymity, confidentiality, safety and the principle of ‘do no harm’. The first author conducted face-to-face interviews in Shona, the local language, at participants’ homes or TVET centres.

### Data analysis

Before data analysis, interview recordings were transcribed verbatim into Shona. In the second round of the process, English translations were integrated directly beneath each question and answer. Two native Shona-speaking research assistants carried out this work and cross-reviewed each other’s transcripts to ensure linguistic precision. As a final quality assurance measure, the first author – a native Shona speaker who conducted the interviews – reviewed all completed transcriptions and translations.

Data analysis followed the five steps of IE: (1) formulating the research problem by examining everyday events to identify features of institutional matrices; (2) finding traces of institutional relations, practices, policies, rules and representatives in accounts; (3) seeking interpretations, disconnections, consistencies, differences, tensions and contradictions; (4) examining traces of what is happening in institutions; and (5) mapping ruling relations among people, policies and processes; and writing participants’ experiences central to the study.

To operationalise these steps, we used the Listening Guide – which ‘is a relational, voice-centred method of analysing qualitative research data’ – according to Petrovic et al. (2015). The Listening Guide elevates the voices of traditionally marginalised populations (Woodcock 2016) and aligns with institutional ethnographic principles. Both approaches start from the individual’s standpoint when analysing data – in this study, that of youth with disabilities. The process involved four listening steps: (1) listening to the plot; (2) constructing *I* poems; (3) listening to contrapuntal voices; and (4) composing an analysis (Gilligan et al. 2003).

The first round of listening focused on the plots or themes that emerged from the participants’ accounts, and these were entered into the first column in the consolidated data analysis template. The second listening involved extracting all instances in which the participant used ‘I’, ‘my’, ‘me’ and ‘myself’; these texts were used to isolate their lived experiences, and these were entered into column two of the template. The same themes and ‘I’ texts were then used for the third and fourth rounds of listening, to trace how participants’ ‘I’ focused narrated experiences interacted with the institutional arrangements (policies, rules, regulations, etc.) at the TVET school, family, wider community and national and international levels, and these were also captured in the same template as stages (column) three and (column) four of the listening process. Steps three and four explore how themes interact (Woodcock 2016) by identifying the relationships between themes, thereby producing narratives (Gilligan et al. 2003). From this process, traces of how institutions influenced the participants’ experiences were determined. Using the consolidated analysis template with organised data for all participants, the first author and two research assistants used the deductive approach to collaboratively identify themes from the ‘I’ quotes and used different colours to label similar themes. Consensus on coding and differences in interpretation were discussed and resolved during this process.

### Positionality and reflexivity

The first author occupies a dual positionality: as an academic researcher and professional within Zimbabwe’s TVET sector. Although active in this field, the author remained professionally independent of the consortium of NGOs that funded the ‘TVET for All’ project. The co-authors provided a supervisory and advisory lens, offering critical distance from the field. Also, regular peer debriefing sessions were held with co-authors, who are experts in disability rehabilitation and occupational therapy, to reduce bias and ensure the credibility of the findings. The purposive selection of these two centres was guided by a professional objective to examine established disability-inclusive practices in a low-resource setting. To mitigate potential practitioner bias, the first author employed a reflexive strategy during the deductive coding phase. Two independent research assistants – not involved in data collection – were hired to code the data. The first author and two research assistants reviewed the coded data collaboratively, ensuring that the generated themes and ‘I’ poems accurately reflected the participants’ authentic voices rather than the researcher’s professional preconceptions.

### Ethical considerations

Ethical clearance to conduct this study was obtained from the Stellenbosch University Health Research Ethics Committee (reference number S22/05/080) and the Medical Research Council of Zimbabwe (MRCZ/A/3032).

## Results

The findings presented here capture participants’ experiences and perspectives, which were classified into nine analytic categories across six main themes: entry qualifications and prior education, pedagogical approaches, social integration and peer relationships, external and environmental support, outcomes and career pathways, and policy. Table 3 summarises these categories and themes.

**TABLE 3:** Overview of findings on participants’ experiences and perspectives.

Themes	Analytic categories
Entry qualifications and prior education	Entry qualifications Matching learners with disabilities and existing vocational courses
Pedagogic approaches	Relationship with instructors and pedagogic approaches
Social integration and peer relationships	Relationship with peers and integration into the inclusive learning environment
External and environmental support	Availability and affordability of assistive technology Accessibility of the physical environment and care facilities Family support
Outcome and career pathways	Disability-inclusive employment practices and amplifying the stories of successful graduates with disabilities.
Policy	Government policy

### Entry qualifications and prior education

#### Entry qualifications

Some youth with disabilities were excluded from the programme because they could not read or write:

‘Yes, because when we were selected for these programmes, some did not qualify because they could not read or write, yet they had disabilities.’ (L04, female, physical impairment)

This illustrates how enrolment and participation in basic education shape and expand future opportunities – including participation in TVET – underscoring the importance of ensuring that people with disabilities have access to basic education.

Therefore, achieving accessibility in schools, including vocational schools, should be considered part of a broader set of reforms, rather than an isolated effort to promote inclusion in TVET.

Families sometimes doubt schools’ ability to care for their children with disabilities, leading them to avoid TVET enrolment to protect them. Some TVET centres were also unwelcoming, citing limited capacity to support learners with certain impairments. Sadly, impairment itself is perceived as the barrier, rather than the lack of institutional support.

#### Matching learners with disabilities and existing vocational courses

Assessing learners with disabilities and matching them to suitable vocations is a common practice in inclusive TVET programmes:

‘Besides ramps, the school ensured that learners with disabilities enrolled in courses in line with their impairment.’ (L02, male, physical impairment)

In the assessment and matching processes, TVET centres consider learners’ passions and their employment and work-based learning contexts:

‘Since it takes a long time to change industry facilities, we prefer to screen learners at enrolment and guide them to courses they can participate in without challenges. We also consider their passion.’ (SP02, male, TVET centre B)

However, some participants highlighted the importance of allowing youth with disabilities the freedom to choose their own courses and supporting those choices:

‘I love cooking. When I went to the training centre, I expected to just spend time there, but what I witnessed sparked a passion in me to succeed in life.’ (L09, female, physical impairment)

Others emphasised the importance of family support in their vocational course choices, suggesting that the influence of those conducting the assessment may sometimes constrain their freedom to pursue their preferred career paths:

‘Let us say a young person with disability chooses her own course to pursue. They [*her family*] should source money to pay for it because she [*the young person with a disability*] is confident she can succeed in her preferred course.’ (L08, female, speech impairment)

### Pedagogic approaches

#### Relationship with instructors and pedagogic approaches

The influence of instructors and their pedagogical approaches was evident in the interview data. Most participants attributed their positive experiences of the training programme to the support they received from their instructors:

‘The teachers were patient with us. They told us we were all equal despite coming from different backgrounds.’ (L08, female, speech impairment)

Some youth formed strong bonds with their instructors and maintained these relationships long after completing their training:

‘Another factor that contributed to my success was my teacher’s encouragement. She did not discriminate against me, nor did she consider my stature. To this day, we still communicate.’ (L03, male, wheelchair user)

Their positive experiences were also linked to instructors’ use of inclusive and adaptive teaching methods and resources:

‘Information was displayed on the projector, so I had an idea of what was being taught.’ (L06, female, speech and hearing impairment)

Learners with hearing impairments reported that the scarcity of instructors with adequate sign language skills makes it difficult for them to follow the learning process:

‘If teachers trained in formal sign language work with those experienced in informal sign language, everyone will be accommodated.’ (L04, male, speech and hearing impairment)

However, all instructors were trained in inclusive education, and the participants linked their positive experiences and their instructors’ conduct to the disability awareness they gained through the training:

‘The new teachers were first trained on inclusive education, which encompasses persons with disabilities.’ (L10, male, physical impairment)

The training seems to have strengthened instructors’ understanding of learners with disabilities and shaped how they recognised learners’ needs and interpreted their responsibilities within institutional processes.

### Social integration and peer relationships

#### Relationship with peers and integration into the inclusive learning environment

A welcoming environment and learners’ ability to form relationships with peers without disabilities are crucial to integrating learners with disabilities into inclusive settings. These relationships also often become vital support systems, helping them manage their daily routines at the training centre:

‘When I got there, we were treated the same way; there was no discrimination. That helped us build relationships with others.’ (L09, female, physical impairment)

Successful integration into inclusive learning environments is thus not only a consequence of institutional rules, but also the result of socially coordinated interactions between learners with disabilities and their peers without disabilities. However, some learners with disabilities struggled to build relationships with their peers without disabilities, which slowed their integration:

‘At first, it was a challenge because others looked down upon us; they did not understand us. Those who understood disability encouraged me, but others discouraged me.’ (L02, male, physical impairment)

While some struggles stem from negative interpersonal encounters, others arise from innate fear and uncertainty. Initially, some participants were worried, anxious and unsure whether their peers without disabilities would accept them or whether they would fit into the new inclusive learning environment. Some withdrew and self-isolated to cope with their anxiety:

‘What made me afraid was meeting new people in an unfamiliar environment. Everything was new to me, and I wondered whether I could work with them. But there were good people who helped me.’ (L01, female, physical impairment)

However, for some learners, encouragement from teachers and favourable treatment from supportive peers fostered a growing sense of belonging, which, in turn, continually enabled them to overcome their fears and anxieties:

‘Our teachers encouraged us, telling us we were equal, while others treated us like their own little sisters.’ (L04, female, physical impairment)

‘I was lucky to have a friend with whom I shared a room and everything, even food. Over time, we got used to other students and came to regard each other as equals.’ (L04, female, physical impairment)

The school rules of co-existence outlined how all learners were expected to behave and served as a foundation for recognising and protecting the rights of learners with disabilities:

‘The rules of this school guide us on how to live in that place, respecting each other’s views. I think that [*rules*] make others consider the rights of learners with disabilities.’ (L10, male, physical impairment)

In some cases, these rules exceed the guidelines for inclusive learning. This reflects a deeply rooted humanistic pedagogical approach deliberately integrated into schedules, teaching and learning routines, and regular interface meetings among instructors, learners and authorities to create a truly welcoming environment:

‘We use a humanistic approach; we do not just push for grades or skills; we also consider our learners’ socioeconomic situation. We orient our instructors on the humanistic approach and use our pedagogical framework to manage the behaviour of our instructors and learners.’ (SP01, male, TVET centre A)

While these rules were in place, some learners with disabilities felt that they should be incorporated into student contracts to ensure their enforcement in the event of a violation:

‘I think the rules on how students are expected to treat their peers with disabilities must be included in the contract form.’ (L10, Male, Physical impairment)

Despite school rules promoting co-existence, some participants acknowledged that stigma was inevitable – particularly in the early days – owing to learners’ diverse backgrounds. Home-bred myths and stereotypes often carry over into the school environment, creating conflicts between social norms and school rules. Some learners face stigma from peers without disabilities, occasionally resorting to self-isolation:

‘During the first few days, they looked down on us. We would go to the dining hall as a group of people with disabilities, waiting for those without disabilities to enter first, even though everyone was encouraged to enter the hall together.’ (L02, male, physical impairment)

#### Community support

The accounts of some learners illustrate the symbiotic relationship between schools and their surrounding communities, as well as between schools and the home communities of learners with disabilities. They found encouragement from their home communities and from elders in the community around the school, who occasionally came to check on them:

‘Yes, the community [*around the school*], relatives, and friends from my village came to check on us.’ (L02, male, physical impairment)

Negative comments, name-calling and discouragement from the community deter others from enrolling:

‘Some [*youth with disabilities*] might not want to go to school because they hear other people [*community members*] who look down on them, and tell them they cannot do anything.’ (L09, female, physical impairment)

Thus, the lack of self-confidence among youth with disabilities can be linked to the persistent negative messaging they encounter within their communities. However, some participants reported developing inner strength to overcome the stigma and discrimination they faced in their communities, focusing on cultivating a positive self-perception:

‘I grew up having boys as friends because girls discriminated against me, and this encouraged me to do something in life to prove them wrong. I no longer wait to be shunned by people.’ (L07, female, physical impairment)

This account reflects a form of self-resistance that some learners with disabilities have developed as an expression of agency to help them counter stigma. When such people enrol in TVET programmes, they often have the strength to cope with the stigma.

#### Influence of community-based support groups

Some participants confirmed that they were informed about the opportunity to participate in TVET programmes through disability-focused, community-based groups, indicating that youth with disabilities who belong to such groups and platforms are more likely to access opportunities:

‘We started holding inter-group discussions for people with disabilities in my area, which Mr Thomas [*not his real name*] coordinated. One day, he asked us whether anyone wanted to pursue TVET.’ (L04, female, physical impairment)

Some accounts also confirm the usefulness of institutional databases in connecting youth with disabilities to opportunities:

‘I used to attend career guidance sessions at a local state university, where the names of people with disabilities who wanted to attend school were recorded … I was called to the training centre, and my name was found in the database. Information on available courses was sent to my phone. I then chose the course I wanted to pursue.’ (L10, male, physical impairment)

However, the database’s usefulness was limited to the young person’s personal social network, indicating the presence of gatekeepers who sometimes withhold information and prevent it from reaching the grassroots, where youth with disabilities live:

‘Information about most programmes does not reach the grassroots; it just ends at the top. Some keep the information to themselves.’ (L04, female, physical impairment)

### External and environmental support

#### Availability and affordability of assistive technology

Although learners with disabilities who required assistive devices received them as part of their scholarship support, they called for government subsidies and welfare programmes to ensure that assistive technology is both available and affordable:

‘Some people need assistive devices such as wheelchairs, but they are expensive. So, the government should assist or cover half the cost. Some of the parents are not working or cannot afford them.’ (L09, female, physical impairment)

This finding indicates a dire need for government-funded subsidies and welfare programmes to improve the availability and affordability of assistive technology, as financial barriers continue to hinder the participation and mobility of youth with disabilities in TVET.

#### Accessibility of the physical environment and care facilities

The disaggregated data in Table 1 show that long travel distances to TVET centres were common among male and female learners across all age categories, suggesting that geographical accessibility remains a significant constraint for youth with disabilities seeking TVET.

The participants considered the hostels at both institutions accessible. Both had ramps and wide doors, and the toilets were adapted. One centre had classrooms close to the hostels, easing mobility for learners with disabilities. Customised beds for students with disabilities demonstrate deliberate efforts to enhance accessibility:

‘The place we stayed was comfortable. We had everything: beds and mattresses. Our beds were different from others because they allowed wheelchair users to place their wheelchairs beside their beds, whereas those without disabilities used bunk beds.’ (L02, male, physical impairment)

Without suitable transport arrangements, the distance from the bus stop to the school is another barrier to accessibility for learners with disabilities at one of the two centres:

‘The distance of about 2 km, between the bus stop and the school is very long, especially for those who cannot walk properly.’ (L01, female, physical impairment)

One participant also linked the lack of inclusive or specialised schools to the denial of civic identity among youth with disabilities, thereby hampering their access to TVET.

Without such schools, families often lack the motivation to obtain civil documents, such as birth certificates, required for enrolment in TVET. This illustrates how institutional exclusion leads to broader civic marginalisation:

‘Jairos Jiri [*a special needs school*] is very far from us. They [*youth with disabilities*] will end up staying at home without even a birth certificate, as people will see no value in them having a birth certificate or a national identity document if they are not going to school anyway.’ (L15, female, physical impairment)

#### Family support

Family support also shapes the experiences of learners with disabilities in inclusive TVET centres. Several participants reported being motivated by their families’ support:

‘My family supported me a lot from the moment they heard the news that I was going to participate in the TVET programme; they did not hesitate to enrol me.’ (L01, female, physical impairment)

Although all learners with disabilities enrolled at the two institutions received scholarships, their families were expected to provide practical learning materials, toiletries, extra food, medical supplies and other support.

Consequently, learners from poorer socioeconomic backgrounds may find it more difficult to meet the financial demands of participating in such programmes:

‘There are some [*youth with disabilities*] whose parents are unemployed and have no income; they [*youth with disabilities*] must be assisted to attend school.’ (L09, female, physical impairment)

When families can afford the necessary provisions, it eases pressure on learners with disabilities, helping them focus on their learning:

‘[*My family*] would provide whatever I wanted. If I am sick, they would send medication and other items.’ (L01, female, physical impairment)

Hence, socioeconomic circumstances outside the TVET centre, especially within the learners’ family environment, shape their capacity to engage in TVET programmes. Family support also assisted youth with disabilities in navigating the demands of the training programmes and sustaining themselves within the school environment:

‘They [*my family*] sent me items for practical projects. I did not lack anything, including books, baking utensils, ingredients, and pocket money, which they provided.’ (L09, female, physical impairment)

To cover these expenses, some learners with disabilities receive assistance from social support networks beyond their immediate families. Thus, a wider support system meant that responsibility was shared, easing the burden on their immediate families and ensuring that the learners had what they needed to participate in the training programme:

‘My relatives helped me with clothes, training materials, and books, which were supposed to be brought from home.’ (L05, female, physical impairment)

### Outcomes and career pathway

#### Disability-inclusive employment practices

While enrolling in an inclusive TVET programme is a significant step towards empowering youth with disabilities to develop requisite skills to improve their livelihoods, barriers such as employers’ negative attitudes and the lack of inclusive workplace facilities limit their participation in internships and their transition to employment:

‘When I am done with school, it will be challenging to get a job because employers look down on us, thinking we cannot do anything.’ (L09, female, physical impairment)

Some participants believed that government policy significantly influences employment practices; hence, they called for policy-driven, disability-inclusive employment practices:

‘The government should assist persons with disabilities to obtain employment. There are persons with disabilities who are educated but were denied employment because of their impairment.’ (L07, female, physical impairment)

#### Amplifying the stories of successful graduates with disabilities

Some participants felt that sharing the success stories of gainfully employed or entrepreneurial TVET graduates with disabilities was an effective way to spread positive messages within communities:

‘They should promote it on social media and upload videos of other students with disabilities who have already participated in the programme to help spread the news.’ (L07, male, physical impairment)

Implementing such measures may help communities realise the potential of youth with disabilities if they are allowed to participate in TVET.

### Policy

#### Government policy

Some participants were aware of the systemic challenges that hindered their participation in mainstream education, including TVET programmes. Their call for government-supported scholarships can be seen as a plea to align policy commitments with resources to address socioeconomic barriers to their participation:

‘They [*the Government*] should uplift us [*youth with disabilities*] through scholarships. Some youth with disabilities are intelligent but lack the money [*to pay fees*].’ (L01, female, physical impairment)

They recognise that the government sets national development priorities and that NGOs are often compelled to align with them:

‘The government should intervene in this and encourage NGOs to develop TVET programmes for people with disabilities.’ (L07, female, physical impairment)

Most participants recognised that their enrolment in this TVET programme was made possible by the collaborative efforts of NGOs and the government:

‘They [*two sponsoring organisations*] provide me with bathing soaps, sanitary wear, and school fees.’ (L06, female, physical impairment)

However, some of them felt that other vulnerable groups (e.g. orphans) received more support from the government than people with disabilities and called for more equitable support for all vulnerable groups:

‘We want the authorities to help others like us attend school. Currently, education assistance appears to favour orphans over people with disabilities.’ (L05, male, physical impairment)

## Discussion

Our study findings highlight the conditions affecting the enrolment and participation of youth with disabilities in inclusive TVET. Institutional ethnographic analysis showed how social institutions, policies, ideologies and societal stereotypes interact in complex ways to influence people’s experiences. These influences are deeply embedded in the psyche of youth with disabilities through persistent negative messaging in TVET centres, communities, families and society. When youth from diverse backgrounds converge in vocational centres, some bring harmful myths and stereotypes which often conflict with institutional rules and regulations, creating disconnections in inclusive TVET environments.

### Entry qualifications and prior education

Although the two sampled TVET institutions did not specify entry requirements, some youth with disabilities were disqualified for not having the required literacy level, highlighting the role of gatekeepers involved in mobilisation who often impose extra eligibility rules that affect who qualifies. Thus, lack of basic education generally increases the likelihood of exclusion from TVET – aligning with existing evidence – highlighting how low levels of educational attainment create and sustain social exclusion of persons with disabilities (Sparkes 1999). Evidence from comparable contexts is similar to this finding. Ebuenyi et al. (2020), Melesse, Haley and Wärvik (2022) and Murgor et al. (2014) identify strict admission requirements – that many youth with disabilities do not possess – as a structural barrier to their enrolment and participation in TVET. This social exclusion is reflected in their significant under-representation in TVET, particularly in low-income countries (International Labour Organization 2017).

### Matching learners with disabilities and existing courses

Enrolling in a passion-aligned course generally increases the success rates of TVET graduates. However, youth with disabilities often face limited options because of assessments and matching exercises, which often give the families, schools and experts the power to decide on courses. One participant’s account illustrates how these matching exercises influence enrolment and portrays the impairment as a barrier to choosing a course, rather than focusing on the institutional support required to facilitate participation in preferred courses. While other studies emphasise the importance of pre-enrolment assessments, these assessments must consider the personal interests of youth with disabilities (Adhikari 2018; Bechange et al. 2021; Ebuenyi et al. 2020; Malle et al. 2015; Nuri et al. 2012; Olujide & Ademokoya 2006; Shatil et al. 2023; Vlachos 2008) and involve the input of their families (Vlachos 2008). Studies also emphasise the need to complement these assessments with efforts to customise training programmes and pedagogic approaches to the unique needs of youths with diverse learning needs (Adhikari 2018; Kisanga et al. 2018; Mosalagae 2023; Murgor et al. 2014; Vlachos 2008).

### Pedagogic approaches and relationships with instructors

Turning to pedagogical and institutional practices, the availability of competent instructors in inclusive education is a crucial facilitator of inclusion (Bhilitane et al. 2024; Ebuenyi et al. 2020; Malle et al. 2015; Phuthi & Mazarire 2024; Vlachos 2008). Findings show that positive instructor conduct and inclusive humanistic pedagogical approaches foster positive experiences for learners with disabilities in inclusive TVET. These approaches, often guided by institutional ideological positions, are communicated through mandatory instructor orientations, mainstream teaching plans, staff recruitment policies, codes of conduct, management structures and curricula. Staff training in disability awareness and inclusive education, conducted by both disability experts and sponsoring partners, often guided by international standards, also influences instructors’ conduct. Thus, arguably, learners with disabilities’ positive experiences result from interactions between micro-, meso- and macro-level rules, ideologies, policies and standards.

However, while short-term staff training helps instructors to understand basic inclusive pedagogy, the lack of sign language interpreters at TVET centres highlights the shortcomings of short-term in-service training in equipping instructors to meet the diverse learning needs of learners with disabilities. This finding aligns with the assertion that instructor capacity limitations affect the effective participation of people with disabilities in TVET (Malle 2016). Cabreros and Barbacena (2024) and Malle (2016) argue that instructors frequently lack both pedagogical training and industrial experience, emphasising that short-term orientation is insufficient.

These arguments underscore the need to complement short-term orientation with long-term pre-service or in-service staff training programmes that empower training providers to better support and improve learning outcomes (Rooney 2016).

### Integrating into the new inclusive learning environment

The initial days in an inclusive learning environment are crucial for the social integration of learners with disabilities. While building peer relationships is vital, the stigma, discrimination and anxiety caused by uncertainties in the new environment are often overwhelming. Findings show that some cope, others withdraw and self-isolate, thus affecting their ability to build peer relationships. This finding is similar to Adhikari’s (2018) assertion that a complex interplay of cultural beliefs, attitudes and myths about disability makes it difficult for youth with disabilities to socialise and integrate into inclusive learning environments. Phuthi and Mazarire (2024) also found that learners’ initial negative interpersonal encounters with peers without disabilities, often marked by feelings of uncertainty and inferiority, overwhelm them and hinder their integration into new inclusive settings.

Findings also show that stigma persists, despite school rules promoting co-existence and non-discrimination, underscoring how social norms from learners’ diverse backgrounds can override institutional rules. The instructors’ efforts to combat stigma revealed the conflict between regulations and societal norms in inclusive TVET spaces. However, care and support from instructors and peers foster a sense of belonging and encourage learners with disabilities. Regular meetings between instructors and students, emphasising co-existence and respect, contribute to increased disability awareness and improve attitudes within the TVET centre.

### Community and family support

Jones, Muz and Yadete (2021) and Malle (2016) argue that negative parental attitudes and societal stigmatisation often lead to youth being confined to their homes and excluded from school. Study findings confirm this assertion and show how family and community involvement – in the form of moral support and encouragement – is crucial. Although TVET centres rely on family support, some families cannot afford the costs of training. This partly explains the under-representation of youth with disabilities in TVET in LMICs, where government subsidies are either limited or non-existent. This finding aligns with earlier research (Järnhammer et al. 2018; Nuri et al. 2012), highlighting obstacles arising from the mismatch between families’ socioeconomic situations and the cost of TVET. Yego, Ontumbi and Situma (2025) and Im-motna (2025) aver that even when tuition is covered, the financial burden of providing other training requirements – such as transportation and assistive technology – discourages enrolment and participation.

Community support and encouragement motivated learners with disabilities to work hard and succeed. Conversely, society’s framing of disability as an inability also influences self-beliefs and how they perceive the potential opportunities available to them, including opportunities to participate in TVET. These findings are similar to those of Bhilitane et al. (2024), Ebuenyi et al. (2020), Malle et al. (2015). Society’s framing of disabilities, often linked to medical reports that describe individuals’ impairments, is often used to assign social identities. Some youth with disabilities develop inner strength through self-resistance and agency to face this stigma and discrimination, and when they enrol in TVET, they often overcome these barriers and achieve their goals.

### Accessibility of the physical environment

Accessible infrastructure facilitates enrolment, effective participation, and positive learner experiences (Bhilitane et al. 2024; Kisanga et al. 2018). This supports our study’s findings, which emphasise the importance of accessibility. Most participants concurred that accessibility encompassed the availability of inclusive TVET schools within a reasonable distance from their homes, disability-friendly boarding facilities, proximity of classrooms to training workshops and hostels, provision of disability-friendly transport services, reasonable accommodations and adaptations.

### The influence of community-based support groups

Most participants attributed their entry into TVET to social networks linked to community-based disability support groups, emphasising such networks’ role as key gateways to educational opportunities. Others also credited local NGOs, underscoring their critical role in facilitating access to TVET for youth with disabilities. Thus, youth with disabilities linked to these groups are more likely to get information about educational opportunities – consistent with earlier research asserting that social capital creates opportunities for youth with disabilities, whereas a lack of it creates obstacles (Trainor 2008).

### Government policy and participation in work-based learning

Participants called for better policy and funding alignment, reflecting their low confidence in government support for participation in TVET programmes. While NGOs fill funding gaps, their interventions are less sustainable because their funding is often limited, short-term and project-based. Participants urged the government to help these organisations expand their inclusive TVET programmes. This recommendation highlights how state policy gaps directly restrict the resource priorities of non-state actors.

Youth with disabilities’ participation in work-based learning – a vital component of TVET – depends on employers’ willingness to make workspaces inclusive and accessible. However, attitudinal, environmental and institutional barriers – reflected in employment policies, practices, and inaccessible infrastructure – limit the participation of youth with disabilities. Despite confidence in their abilities, negative attitudes from employers or coworkers can undermine their success (Mitchell, Cully & Hoff 2023; Morwane, Dada & Bornman 2006). They rank employer misconceptions and a lack of disability knowledge among the main barriers to post-training work transition.

### Limitations

While this study offers nuanced insights into the experiences of learners with disabilities in inclusive TVET programmes, several limitations must be acknowledged. Firstly, its geographical scope is limited to one province and two TVET centres implementing an NGO-supported project, which may reflect a best-case scenario that does not necessarily represent broader regional standards. Secondly, although the study highlights the experiences of learners with disabilities, its findings lack perspectives from other stakeholders, such as instructors, family members and community leaders. Additionally, despite using an IE framework, the study did not include a formal document review, leaving policy influences to be interpreted solely through participant narratives. Future research should therefore incorporate text-based policy analysis to achieve a more comprehensive understanding of the institutional forces affecting inclusion. Despite these limitations, it offers a detailed analysis of the hidden, multi-institutional forces influencing learners’ experiences within inclusive vocational programmes.

## Conclusion

This study highlights the importance of positive instructor conduct, inclusive pedagogies, access to basic education, family and community support, positive societal attitudes, government backing and accessible infrastructure in enabling youth with disabilities to participate in TVET. It further reveals complex, often invisible interactions among societal rules and institutional influences across the entire training cycle, from pre-enrolment to post-training employment, to shape the experiences of learners with disabilities. By adopting a learner-centred approach and an IE lens, the study offers valuable insights for developing inclusive, user-focused TVET programmes and policies in low-resource settings.
